# Publisher Correction: Distinct patterns of within-host virus populations between two subgroups of human respiratory syncytial virus

**DOI:** 10.1038/s41467-021-26291-y

**Published:** 2021-10-07

**Authors:** Gu-Lung Lin, Simon B. Drysdale, Matthew D. Snape, Daniel O’Connor, Anthony Brown, George MacIntyre-Cockett, Esther Mellado-Gomez, Mariateresa de Cesare, David Bonsall, M. Azim Ansari, Deniz Öner, Jeroen Aerssens, Christopher Butler, Louis Bont, Peter Openshaw, Federico Martinón-Torres, Harish Nair, Rory Bowden, RESCEU Investigators, Harry Campbell, Steve Cunningham, Debby Bogaert, Philippe Beutels, Joanne Wildenbeest, Elizabeth Clutterbuck, Joseph McGinley, Ryan Thwaites, Dexter Wiseman, Alberto Gómez-Carballa, Carmen Rodriguez-Tenreiro, Irene Rivero-Calle, Ana Dacosta-Urbieta, Terho Heikkinen, Adam Meijer, Thea Kølsen Fischer, Maarten van den Berge, Carlo Giaquinto, Michael Abram, Philip Dormitzer, Sonia Stoszek, Scott Gallichan, Brian Rosen, Eva Molero, Nuria Machin, Martina Spadetto, Tanya Golubchik, Andrew J. Pollard

**Affiliations:** 1 Oxford Vaccine Group, Department of Paediatrics, University of Oxford, Oxford, UK; 2 NIHR Oxford Biomedical Research Centre, Oxford, UK; 3 Peter Medawar Building for Pathogen Research, University of Oxford, Oxford, UK; 4 Wellcome Centre for Human Genetics, University of Oxford, Oxford, UK; 5 Big Data Institute, Nuffield Department of Medicine, University of Oxford, Oxford, UK; 6 Translational Biomarkers, Infectious Diseases Therapeutic Area, Janssen Pharmaceutica NV, Beerse, Belgium; 7 Nuffield Department of Primary Care Health Sciences, University of Oxford, Oxford, UK; 8 Department of Pediatrics, Wilhelmina Children’s Hospital, University Medical Center Utrecht, Utrecht, Netherlands; 9 ReSViNET Foundation, Zeist, Netherlands; 10 National Heart and Lung Institute, Imperial College London, London, UK; 11 Translational Pediatrics and Infectious Diseases, Hospital Clínico Universitario de Santiago de Compostela, Santiago de Compostela, Spain; 12 Genetics, Vaccines, Infectious Diseases, and Pediatrics Research Group (GENVIP), Instituto de Investigación Sanitaria de Santiago de Compostela, Santiago de Compostela, Spain; 13 Centre for Global Health, Usher Institute, Edinburgh Medical School, University of Edinburgh, Edinburgh, UK; 14 Queen’s Medical Research Institute, University of Edinburgh, Edinburgh, UK; 15 Centre for Health Economics Research and Modelling Infectious Diseases, Vaccine and Infectious Disease Institute, University of Antwerp, Antwerp, Belgium; 16 Department of Pediatrics, University of Turku, Turku University Hospital, Turku, Finland; 17 National Institute for Public Health and the Environment, Bilthoven, Netherlands; 18 Statens Serum Institut, Copenhagen, Denmark; 19 Department of Pulmonary Diseases, University of Groningen, University Medical Center Groningen, Groningen, Netherlands; 20 PENTA Foundation, Padua, Italy; 21 AstraZeneca, Gaithersburg, MD US; 22 Pfizer, Pearl River, NY US; 23 GlaxoSmithKline, Potomac, MD US; 24 Sanofi Pasteur, Toronto, Ontario Canada; 25 Novavax, Potomac, MD US; 26 Team-It Research, Barcelona, Spain; 27 Present Address: Paediatric Infectious Diseases Research Group, Institute for Infection and Immunity, St George’s, University of London, London, UK; 28 Present Address: Division of Advanced Technology and Biology, Walter and Eliza Hall Institute of Medical Research, Melbourne, VIC Australia

**Keywords:** Viral genetics, Viral evolution, Viral infection

Correction to: *Nature Communications* 10.1038/s41467-021-25265-4, published online 26 August 2021.

The original HTML version of this Article contained errors in the author affiliations, which were correct in the PDF version. Specifically, affiliations after number 14 in the PDF version were displaying incorrectly in the online version of the paper. This has been updated so that all affiliations are accurate in the HTML version of the Article.

