# Supplementary material for: Distinct patterns of within-host virus populations between two subgroups of human respiratory syncytial virus
Source: Nat Commun. 2021 Aug 26;12:5125. doi: 10.1038/s41467-021-25265-4 (PMC8390747; doi:10.1038/s41467-021-25265-4)
Supplement: Supplementary file 1 — Supplementary Information [file 41467_2021_25265_MOESM1_ESM.pdf]

Supplementary Information

Distinct patterns of within-host virus populations between two subgroups of human respiratory syncytial virus

Gu-Lung Lin[1,2,*], Simon B Drysdale[1,2,3], Matthew D Snape[1,2], Daniel O'Connor[1,2], Anthony Brown[4], George MacIntyre-Cockett[5], Esther Mellado-Gomez[5], Mariateresa de Cesare[5], David Bonsall[5,6], M Azim Ansari[5], Deniz Öner[7], Jeroen Aerssens[7], Christopher Butler[8], Louis Bont[9,10], Peter Openshaw[11], Federico Martinón-Torres[12,13], Harish Nair[14], Rory Bowden[5,15], RESCEU Investigators[a], Tanya Golubchik[6], and Andrew J Pollard[1,2]

[1]Oxford Vaccine Group, Department of Paediatrics, University of Oxford, Oxford, UK.
[2]NIHR Oxford Biomedical Research Centre, Oxford, UK.
[3]Present address: Paediatric Infectious Diseases Research Group, Institute for Infection and Immunity, St George's, University of London, London, UK.
[4]Peter Medawar Building for Pathogen Research, University of Oxford, Oxford, UK.
[5]Wellcome Centre for Human Genetics, University of Oxford, Oxford, UK.
[6]Big Data Institute, Nuffield Department of Medicine, University of Oxford, Oxford, UK.
[7]Translational Biomarkers, Infectious Diseases Therapeutic Area, Janssen Pharmaceutica NV, Beerse, Belgium.
[8]Nuffield Department of Primary Care Health Sciences, University of Oxford, Oxford, UK.
[9]Department of Pediatrics, Wilhelmina Children's Hospital, University Medical Center Utrecht, Utrecht, Netherlands.
[10]ReSViNET Foundation, Zeist, Netherlands.
[11]National Heart and Lung Institute, Imperial College London, London, UK.
[12]Translational Pediatrics and Infectious Diseases, Hospital Clínico Universitario de Santiago de Compostela, Santiago de Compostela, Spain.
[13]Genetics, Vaccines, Infectious Diseases, and Pediatrics Research Group (GENVIP), Instituto de Investigación Sanitaria de Santiago de Compostela, Santiago de Compostela, Spain.
[14]Centre for Global Health, Usher Institute, Edinburgh Medical School, University of Edinburgh, Edinburgh, UK.
[15]Present address: Division of Advanced Technology and Biology, Walter and Eliza Hall Institute of Medical Research, Melbourne, Australia.
[a]A list of authors and their affiliations appears at the end of the paper.
These authors jointly supervised this work: Tanya Golubchik, Andrew J Pollard.
[*]E-mail: gu-lung.lin@paediatrics.ox.ac.uk

Supplementary Table 1 Characteristics of RSV samples by sequencing batch.[a]

	Batch 1 (N = 17)	Batch 2 (N = 374)	Batch 3 (N = 94)	Batch 4 (N = 373)
Included samples for this study	11	113	41	157
RSV-A samples	3	53	16	104
RSV-B samples	8	60	25	53
From infants	11	106	39	157
From older adults	0	7	2	0
Sequencing platform	MiSeq	NovaSeq	MiSeq	NovaSeq
Read length	265	151	300	151
Raw read pairs ($\log_{10}$)[b]	6.11 (5.51–6.73)	6.09 (5.44–7.36)	5.39 (4.96–6.43)	6.14 (5.15–7.47)
Unique RSV read pairs ($\log_{10}$)[b]	5.13 (4.13–5.65)	4.57 (4.02–5.91)	4.40 (4.01–5.52)	4.73 (4.01–5.76)
Percent duplication[b]	78 (50–91)	91 (80–98)	73 (37–88)	91 (72–98)
Minimum genome coverage (%)	100	100	100	99.9
Depth of coverage[b]	3,763 (696–7,601)	3,561 (1,091–7,930)	2,209 (525–7,157)	3,994 (719–7,897)

[a] Sequencing statistics were based on the included samples only.
[b] Data are shown as median (range).

Supplementary Table 2 A multiple linear regression model to evaluate the association between pairwise nucleotide diversity and other variables.[a]

Variable	Coefficient	Standard error	T value	P value[b]
Intercept	1.2×10^{-3}	8.1×10^{-5}	15.20	$< 2 \times 10^{-16}$
Country				
Spain (reference)				
Netherlands	-1.3×10^{-5}	1.7×10^{-5}	−0.77	0.44
United Kingdom	8.1×10^{-6}	1.4×10^{-5}	0.57	0.57
Season				
2017–18 (reference)				
2018–19	1.2×10^{-5}	2.1×10^{-5}	0.60	0.55
2019–20	3.0×10^{-5}	3.6×10^{-5}	0.83	0.41
Subgroup				
RSV-A (reference)				
RSV-B	2.3×10^{-5}	1.1×10^{-5}	2.02	0.044
Batch				
First (reference)				
Second	-3.3×10^{-4}	3.6×10^{-5}	−9.11	$< 2 \times 10^{-16}$
Third	-3.3×10^{-4}	3.9×10^{-5}	−8.58	4.8×10^{-16}
Fourth	-5.3×10^{-4}	4.5×10^{-5}	−11.62	$< 2 \times 10^{-16}$
RSV read ($\log_{10}$)	1.1×10^{-5}	1.3×10^{-5}	0.83	0.41
Age group				
Older adult (reference)				
Infant	-1.2×10^{-4}	3.5×10^{-5}	−3.45	0.0006
Severity[c]				
Mild (reference)				
Moderate	1.1×10^{-5}	1.2×10^{-5}	0.94	0.35
Severe	1.7×10^{-5}	1.6×10^{-5}	1.03	0.30

[a] $F(12, 306) = 46.5$, $P < 2.2 \times 10^{-16}$, $R^2 = 0.65$, adjusted $R^2 = 0.63$.

[b] Two-tailed t-tests.

[c] Thirteen patients had missing information on disease severity. These missing data were imputed using the aregImpute function, implemented in the R package Hmisc.

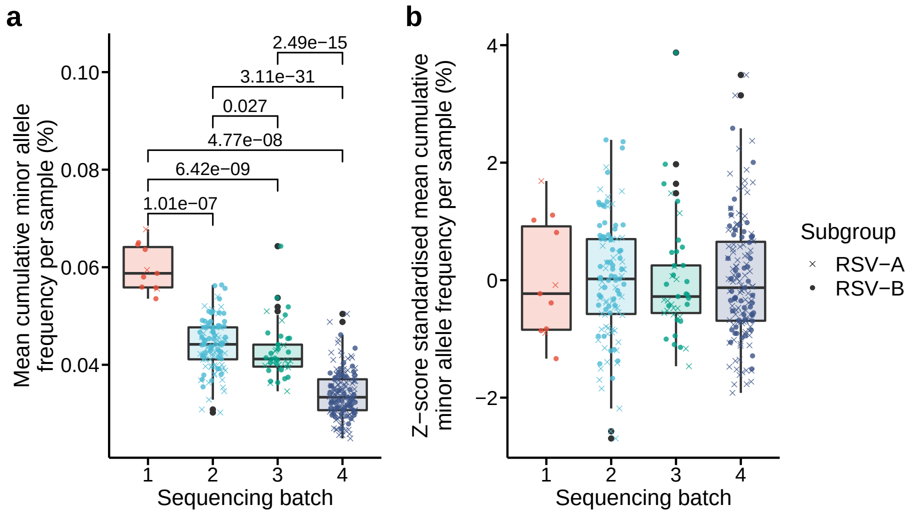

Supplementary Fig. 1 Adjusting for batch effects on cumulative minor allele frequency. **a** Before adjustment. **b** After z-score standardisation of the data. Each dot represents an individual sample. There were 11, 112, 41, and 155 samples from each batch respectively. Two-tailed Mann–Whitney U tests with the Benjamini–Hochberg procedure were used to evaluate the significance of the batch effects. Adjusted P values of less than 0.05 are shown above the box plots. The centre line of each box denotes the median; box limits, the first and third quartiles; whiskers, the highest and lowest values within 1.5 times the interquartile range from the box limits; and outlying points, outliers.

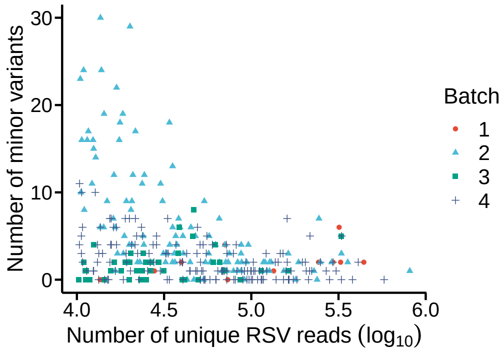

Supplementary Fig. 2 Correlation between the number of minor variants and the number of unique RSV reads. There were a total of 319 samples, with 11, 112, 41, and 155 from each batch respectively. Among the low-burden samples (i.e., those with $\leq 4.5 \log_{10}$ uniquely mapped reads), there were 25 samples in batch 2 having >10 minor variants. Overall, samples in batch 2 had a higher mean unadjusted minor allele frequency (MAF) per sample than batches 3 and 4, as we have shown in Supplementary Fig. 1a. Therefore, the higher mean MAF per sample in batch 2, together with a greater variance of MAF in low-burden samples, caused a greater number of minor variants in some low-burden samples in batch 2. The samples with the greatest numbers of minor variants in this batch represented both subgroups, with 11 RSV-A and 14 RSV-B, contributing similarly to diversity in both subgroups.

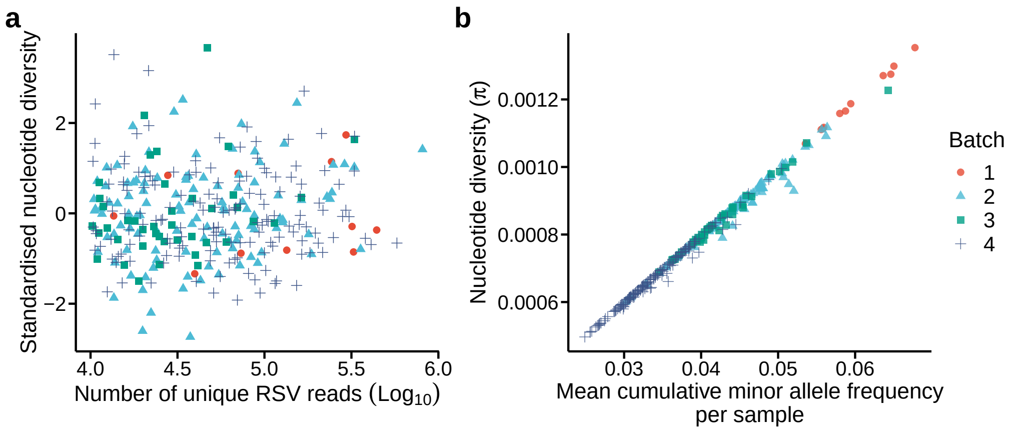

Supplementary Fig. 3 Correlation between pairwise nucleotide diversity and other variables. a Correlation between z-score standardised pairwise nucleotide diversity and the number of unique RSV reads (Pearson correlation analysis, r = 0.053, P = 0.34, two-tailed). **b** Correlation between unadjusted pairwise nucleotide diversity and the mean cumulative minor allele frequency per sample (Pearson correlation analysis, r = 0.997, $P < 2.2 \times 10^{-16}$, two-tailed). Each dot represents an individual sample, annotated by sequencing batch. There were a total of 319 samples, with 11, 112, 41, and 155 from each batch respectively.

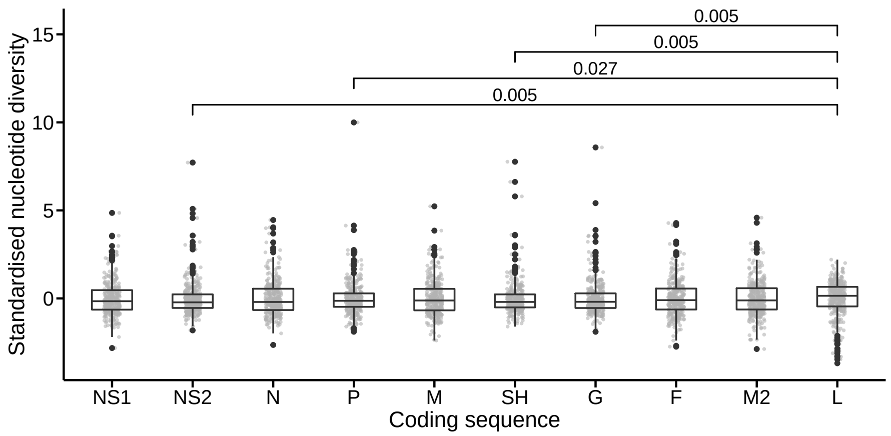

Supplementary Fig. 4 Gene-wise comparisons of z-score standardised pairwise nucleotide diversity. Each dot represents an individual sample. There were a total of 319 samples. Pairwise two-tailed Mann–Whitney U tests with the Benjamini–Hochberg procedure were used to evaluate the differences between each gene. Adjusted P values of less than 0.05 are shown above the box plots. The centre line of each box denotes the median; box limits, the first and third quartiles; whiskers, the highest and lowest values within 1.5 times the interquartile range from the box limits; and outlying points, outliers.

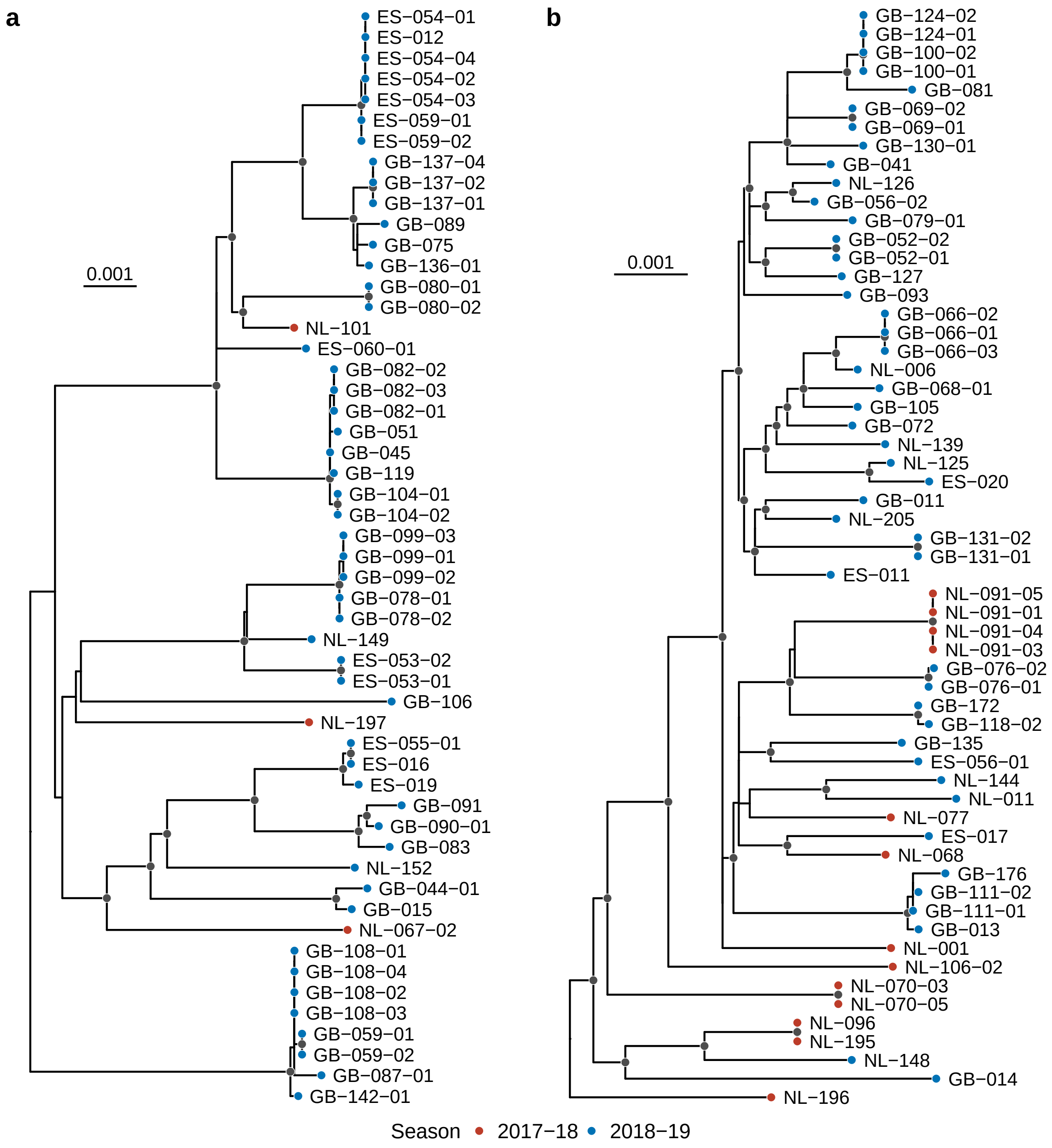

Supplementary Fig. 5 Maximum-likelihood phylogenies of consensus coding sequences of RSV-A (a) and RSV-B (b). These phylogenies only included samples from the second sequencing batch (53 RSV-A and 59 RSV-B strains). Taxa are labelled with country (ES, Spain; GB, United Kingdom; and NL, Netherlands), participant ID, and days of hospitalisation if multiple samples were collected. Scale bars show nucleotide substitutions per site. Black dots are well-supported nodes with a bootstrap value of >70%. The phylogenies were rooted to the NCBI reference strains with the accession numbers of NC_038235 and NC_001781 for RSV-A and RSV-B, respectively. The reference strains are not shown here.

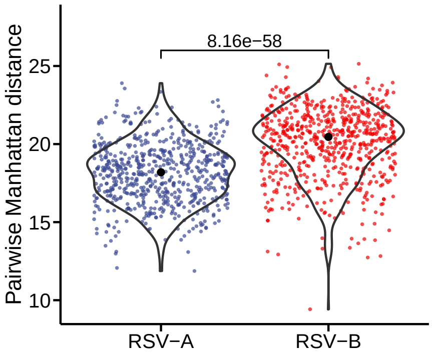

Supplementary Fig. 6 Manhattan distances between sample pairs within the RSV-A and RSV-B subgroups. Original allele frequencies were used to calculate pairwise Manhattan distances. Only pairwise distances between samples from the second sequencing batch, the same country, the same season, and different participants were included. Each coloured dot is a sample pair (650 RSV-A pairs and 656 RSV-B pairs in total). The violin plots summarise the distribution of the data, and the black dots denote the median value of each group. A two-tailed Mann–Whitney U test was used to evaluate the statistical significance of the difference between the subgroups. P value is shown above the violin plots.

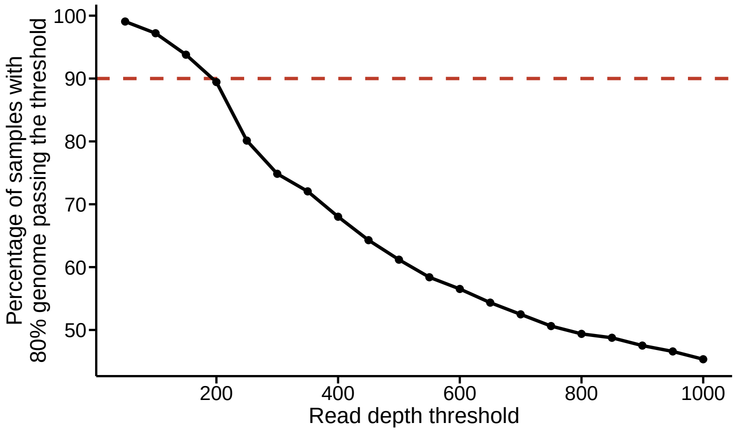

Supplementary Fig. 7 Percentage of the samples with ≥80% of the genomic positions passing each read depth threshold (from 50 to 1,000). Dashed line shows the predefined criterion of 90% of the samples.
