# Supplementary material for: Distinct patterns of within-host virus populations between two subgroups of human respiratory syncytial virus
Source: Nat Commun. 2021 Aug 26;12:5125. doi: 10.1038/s41467-021-25265-4 (PMC8390747; doi:10.1038/s41467-021-25265-4)
Supplement: Supplementary file 3 — Reporting Summary [file 41467_2021_25265_MOESM3_ESM.pdf]

nature research

Corresponding author(s): Gu-Lung Lin

Last updated by author(s): Jul 17, 2021

Reporting Summary

Nature Research wishes to improve the reproducibility of the work that we publish. This form provides structure for consistency and transparency in reporting. For further information on Nature Research policies, see Authors & Referees and the Editorial Policy Checklist .

Statistics

For all statistical analyses, confirm that the following items are present in the figure legend, table legend, main text, or Methods section.

n/a	Confirmed	
☐	☒	The exact sample size (*n*) for each experimental group/condition, given as a discrete number and unit of measurement
☐	☒	A statement on whether measurements were taken from distinct samples or whether the same sample was measured repeatedly
☐	☒	The statistical test(s) used AND whether they are one- or two-sided<br>*Only common tests should be described solely by name; describe more complex techniques in the Methods section.*
☐	☒	A description of all covariates tested
☐	☒	A description of any assumptions or corrections, such as tests of normality and adjustment for multiple comparisons
☐	☒	A full description of the statistical parameters including central tendency (e.g. means) or other basic estimates (e.g. regression coefficient) AND variation (e.g. standard deviation) or associated estimates of uncertainty (e.g. confidence intervals)
☐	☒	For null hypothesis testing, the test statistic (e.g. *F*, *t*, *r*) with confidence intervals, effect sizes, degrees of freedom and *P* value noted<br>*Give P values as exact values whenever suitable.*
☒	☐	For Bayesian analysis, information on the choice of priors and Markov chain Monte Carlo settings
☒	☐	For hierarchical and complex designs, identification of the appropriate level for tests and full reporting of outcomes
☐	☒	Estimates of effect sizes (e.g. Cohen's *d*, Pearson's *r*), indicating how they were calculated

Our web collection on statistics for biologists contains articles on many of the points above.

Software and code

Policy information about availability of computer code

Data collection	No software was used.
Data analysis	Trimmomatic (version 0.39): adapter and quality trimming<br>IVA (version 1.0.8) and SPAdes (version 3.14.1): de novo assembly<br>MAFFT (version 7.471): multiple sequence alignment<br>BLASTN (version 2.7.1+): read and contig classification<br>shiver (downloaded on 2020-08-13): genome reconstruction<br>Bowtie 2 (version 2.4.1): read alignment<br>MarkDuplicates (Picard tools, version 2.18.14): deduplication<br>RAxML (version 8.2.12): maximum-likelihood phylogenetic analysis<br>R (version 4.0.2): data management, statistical analyses, and figure production<br>ggtree (version 2.2.4): an R package for phylogenetic tree visualisation<br>ape (version 5.4-1): an R package for pairwise patristic distance calculation<br>Hmisc (version 4.5-0): an R package for missing data imputation

For manuscripts utilizing custom algorithms or software that are central to the research but not yet described in published literature, software must be made available to editors/reviewers. We strongly encourage code deposition in a community repository (e.g. GitHub). See the Nature Research guidelines for submitting code & software for further information.

Data

Policy information about availability of data

All manuscripts must include a data availability statement. This statement should provide the following information, where applicable:

- Accession codes, unique identifiers, or web links for publicly available datasets
- A list of figures that have associated raw data
- A description of any restrictions on data availability

The sequencing read data generated in this study have been deposited in the European Nucleotide Archive under study accession PRJEB34042 (https://www.ebi.ac.uk/ena/data/view/PRJEB34042). The RSV genomic sequences generated in this study have been deposited in GenBank under accession numbers LR699315, LR699726, LR699734, LR699736–LR699744, and MZ515551–MZ516143 (https://www.ncbi.nlm.nih.gov/nuccore/?term=LR699315%5BPACC%5D+OR+LR699726%5BPACC%5D+OR+LR699734%5BPACC%5D+OR+LR699736%3ALR699744%5BPACC%5D+OR+MZ515551%3AMZ516143%5BPACC%5D). The RSV reference sequences used in this study are available in GenBank under accession numbers NC_038235 (https://www.ncbi.nlm.nih.gov/nuccore/NC_038235) and NC_001781 (https://www.ncbi.nlm.nih.gov/nuccore/NC_001781). The associated sample and de-identified clinical information used in this study is provided in the Supplementary Information file.

Field-specific reporting

Please select the one below that is the best fit for your research. If you are not sure, read the appropriate sections before making your selection.

☒ Life sciences ☐ Behavioural & social sciences ☐ Ecological, evolutionary & environmental sciences

For a reference copy of the document with all sections, see nature.com/documents/nr-reporting-summary-flat.pdf

Life sciences study design

All studies must disclose on these points even when the disclosure is negative.

Sample size	All RSV-positive samples (N = 858) collected from the REspiratory Syncytial virus Consortium in EUrope (RESCEU) project during the 2017–20 RSV seasons were sequenced for this study. Samples fulfilling the inclusion criteria of the within-host virus analysis were included in this study (N = 322). After removing three outlier samples, within-host genetic diversity of RSV was characterised from a total of 319 samples (44% RSV-A and 36% RSV-B), which represent the most comprehensive dataset to date.
Data exclusions	There was one RSV-A and two RSV-B samples with a significantly higher mean cumulative minor allele frequency (MAF) per sample, 0.52%, 0.17%, and 0.19% respectively, than that of all other samples (mean 0.039%; range 0.025%–0.068%). These three samples were excluded from the within-host diversity analysis because they presumably represented a real or artefactual mixture of genetically distinct strains of the same RSV subgroup. In addition, genomic positions with read depth of less than 200 were excluded from the calculations of mean cumulative MAF per sample, nucleotide diversity, and Manhattan distances because sites with low read depth had a greater variance of minor variants than sites with high read depth due to a small sampling fraction. These exclusions were based on a pre-established analysis plan.
Replication	Our study was based on 858 RSV samples (i.e., biological replicates). Among them, 319 RSV samples that generated enough RSV reads with a single RSV subgroup were included in our analyses (excluding three samples that had a significantly high mean cumulative minor allele frequency per sample). The findings presented in the manuscript were summarised from these 319 replicates.
Randomization	All available samples were sequenced and analysed, so no randomisation was performed. Sequencing was done in four different batches given the large number of samples in this study, and multiple linear regression and z-score standardisation of the data were applied to control for the covariate (i.e., batch).
Blinding	Investigators were not blinded to group allocation during data collection and analysis. Blinding was not relevant to our study as there is no group allocation involved in this study.

Reporting for specific materials, systems and methods

We require information from authors about some types of materials, experimental systems and methods used in many studies. Here, indicate whether each material, system or method listed is relevant to your study. If you are not sure if a list item applies to your research, read the appropriate section before selecting a response.

Materials & experimental systems

n/a	Involved in the study	
☒	☐	Antibodies
☒	☐	Eukaryotic cell lines
☒	☐	Palaeontology
☒	☐	Animals and other organisms
☐	☒	Human research participants
☐	☒	Clinical data

Methods

n/a	Involved in the study	
☒	☐	ChIP-seq
☒	☐	Flow cytometry
☒	☐	MRI-based neuroimaging

Human research participants

Policy information about studies involving human research participants

Population characteristics	All of the participants (n = 459) who had their sample(s) sequenced had acute RSV infection. Samples collected from 267 participants were included in this within-host diversity study. 97% (258/267) of them were infants under 1 year of age (median 4.3 months; range 0.2–11.7 months; excluding one infant without the information on age), and 3% (9/267) of them were adults over 60 years of age (median 75 years; range 69–78 years). 43% (110/257) of the infants were female (excluding one without the information on sex), and 78% (7/9) of the adults were female.
Recruitment	Potential adult participants or the parents or guardians of potential infant participants of the clinical studies were approached by study nurses in communities, emergency departments, or hospital wards.<br>Only samples yielding >10,000 RSV deduplicated reads were selected to be included in the within-host virus diversity analysis, which might have introduced a selection bias. Excluding samples with low RSV reads (i.e., viral burden) may have excluded samples collected from patients with certain features. However, the included dataset encompassed patients with different severity of RSV disease, so we were still able to characterise within-host RSV diversity from a wide range of patients.
Ethics oversight	These clinical studies were approved by the relevant ethics committees at each site, including the University of Oxford, the Health Research Authority (IRAS IDs: 224156 and 231136), the NHS National Research Ethics Service Oxfordshire Committee A (reference number: 15/SC/0335), the South Central and Hampshire A Research Ethics Committee (reference number: 17/SC/0522), and the London—Central Research Ethics Committee (reference number: 17/LO/1210) in the UK; Hospital Clínico Universitario de Santiago de Compostela, and Comité de Ética de la Investigacíon de Santiago-Lugo (reference number: 2017/395) in Spain; the Medical Ethical Committee, University Medical Center Utrecht (reference number: 17/563), and the Ethical Review Authority (reference number: NL60910.041.17) in the Netherlands.

Note that full information on the approval of the study protocol must also be provided in the manuscript.

Clinical data

Policy information about clinical studies

All manuscripts should comply with the ICMJEguidelines for publication of clinical research and a completedCONSORT checklist must be included with all submissions.

Clinical trial registration	Birth cohort study: NCT03627572<br>Infant case–control study: NCT03756766<br>Adult cohort study: NCT03621930
Study protocol	Birth cohort study: https://doi.org/10.1093/infdis/jiaa310<br>Infant case–control study: https://doi.org/10.1093/infdis/jiaa239<br>Adult cohort study: https://doi.org/10.1183/13993003.02688-2020
Data collection	This is not a randomised controlled trial. The participants' information and samples were collected at participants' house, emergency departments, or hospital wards in Santiago de Compostela, Spain; in London and Oxford, the UK; and in Utrecht, the Netherlands during the 2017–2020 RSV seasons.
Outcomes	Genetic characteristics of RSV are one of the predefined secondary outcomes of the clinical studies. Next-generation sequencing of RSV samples on the Illumina MiSeq and NovaSeq platforms and bioinformatic analysis of the sequencing data were carried out to assess these measures.
