# Supplementary material for: Distinct patterns of within-host virus populations between two subgroups of human respiratory syncytial virus
Source: Nat Commun. 2021 Aug 26;12:5125. doi: 10.1038/s41467-021-25265-4 (PMC8390747; doi:10.1038/s41467-021-25265-4)
Supplement: Supplementary file 4 — Description of Additional Supplementary Files [file 41467_2021_25265_MOESM4_ESM.pdf]

File Name: Supplementary Data 1

Description: This file lists all samples sequenced in this study with associated accession numbers of the raw sequencing reads and assembled consensus sequences. De-identified participant, sample, sequencing, and clinical information is also provided in this file. Samples included in the within-host virus diversity analysis are annotated under the ‘Included_in_this_study’ column.
